# Effectiveness of Neuraminidase Inhibitors for Preventing Staff Absenteeism during Pandemic Influenza 

**DOI:** 10.3201/eid1303.060309

**Published:** 2007-03

**Authors:** Vernon J. Lee, Mark I. Chen

**Affiliations:** * Tan Tock Seng Hospital, Singapore

**Keywords:** influenza, modeling, absenteeism, healthcare worker, treatment, prophylaxis, policy, pandemic, research

## Abstract

We used a deterministic SEIR (susceptible-exposed-infectious-removed) meta-population model, together with scenario, sensitivity, and simulation analyses, to determine stockpiling strategies for neuraminidase inhibitors that would minimize absenteeism among healthcare workers. A pandemic with a basic reproductive number (R_0_) of 2.5 resulted in peak absenteeism of 10%. Treatment decreased peak absenteeism to 8%, while 8 weeks’ prophylaxis reduced it to 2%. For pandemics with higher R_0_, peak absenteeism exceeded 20% occasionally and 6 weeks’ prophylaxis reduced peak absenteeism by 75%. Insufficient duration of prophylaxis increased peak absenteeism compared with treatment only. Earlier pandemic detection and initiation of prophylaxis may render shorter prophylaxis durations ineffective. Eight weeks’ prophylaxis substantially reduced peak absenteeism under a broad range of assumptions for severe pandemics (peak absenteeism >10%). Small investments in treatment and prophylaxis, if adequate and timely, can reduce absenteeism among essential staff.

## Abstract 2

Timely and adequate treatment and prophylaxis may reduce absenteeism among healthcare workers during the peak of a pandemic.

Concerns regarding the advent and impact of the next influenza pandemic have led >120 countries to develop pandemic preparedness plans (*1*). Studies have shown that treatment with neuraminidase inhibitors and prophylaxis of selected subpopulations are cost-effective strategies to limit the pandemic’s impact on the healthcare system (*2*,*3*). However, supplies of neuraminidase inhibitors are limited, and countries may not have the financial resources to purchase large stockpiles. Policymakers will thus have to determine priorities for treatment and prophylaxis.

One priority is to maintain essential services during the pandemic’s peak—to ensure business continuity and mitigate the resultant damage. Absenteeism of essential staff from work should be minimized to prevent service disruption when most needed. This is particularly crucial for healthcare workers (HCWs) because they may have an increased risk for exposure and illness while facing a surge in demand for healthcare services.

A recent study proposed that hospitals should consider stockpiling neuraminidase inhibitors for treatment and prophylaxis (*4*). To provide policy guidance to reduce the pandemic’s impact on HCWs, this study analyzed the use of neuraminidase inhibitors in minimizing absenteeism by simulating an HCW population in a transmission dynamics model.

## Methods

### Model Structure and Dynamics

**Figure 1:**
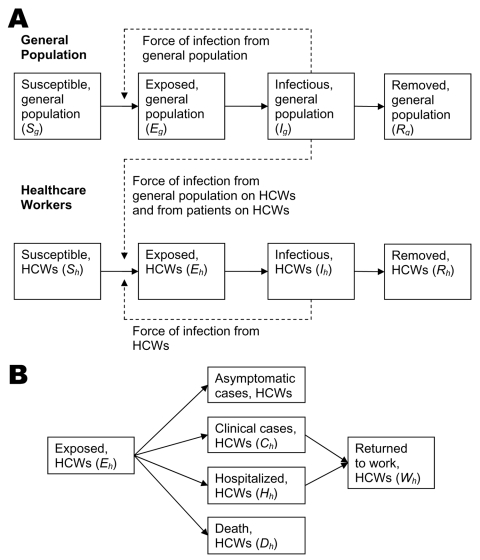
A) Modified SEIR (susceptible-exposed-infectious-removed) model for transmission of pandemic influenza within the general population and healthcare worker (HCW) subpopulation. B) Absenteeism among exposed HCWs.

We used a deterministic, modified SEIR (susceptible-exposed-infectious-removed) meta-population model to evaluate strategies for minimizing absenteeism among HCWs during an influenza pandemic. The model consisted of 2 distinct populations in Singapore: the general population and an HCW population (Figure 1A). Singapore’s mid-year population in 2005 was 4.35 million, and the public HCW population of 20,000 represented essential staff that required protection. Oseltamivir was the neuraminidase-inhibitor modeled because of its effectiveness in treatment and prophylaxis, good safety profile, and common use in national stockpiles (*5*–*8*). Standard treatment regimen was 75 mg, twice per day for 5 days, and prophylaxis required 75 mg once per day for as long as planned.

This study assumed that the general population did not receive treatment or prophylaxis with oseltamivir. Three strategies for HCWs were considered: no action (providing symptomatic relief), treatment only (early treatment of all symptomatic HCW infections), and prophylaxis (prophylaxis together with early treatment). Different predetermined prophylaxis substrategies were considered, based on the weeks of prophylaxis; each additional week required 140,000 doses in addition to separate treatment stockpiles. To be conservative, we assumed that prophylaxis stockpiles would last only for the planned duration. Separate analyses explored the effect of stopping prophylaxis after individual clinical infection, with redistribution of prophylaxis doses to other HCWs to prolong prophylaxis beyond the planned duration; however, this strategy is only possible if tests can promptly confirm individual infection and logistics networks allow for redistribution.

We assumed that all persons were susceptible to the pandemic virus and that the general population epidemic occurred as a single wave after introduction of a single infectious case. We ignored the contribution of new introductions after the start of the epidemic. Persons were removed from the susceptible state, after infection, through recovery or death (Figure 1A). Births, deaths from other causes, immigration, and emigration during the period were assumed to be negligible.

We assumed a range of infectious periods similar to those from other studies; we also assumed that the disease was infectious at about the same time a person became symptomatic; i.e., the latent period coincided with the incubation period (*9*,*10*). A range of basic reproductive numbers (R_0_), based on these infectious and latent periods, were then used to generate epidemics in the general population with varying rates of transmission. These R_0_ then determined the course of the HCW epidemic.

HCWs were assumed to be exposed to influenza from 3 sources and may be more likely to be exposed than the general population (*11*). The first source was exposures from colleagues (HCW-to-HCW transmission) at a proportion (ω); the second was from persons outside the workplace (*1-ω*). In the absence of published estimates, the base case assumed that 50% of infections were attributed to HCW-to-HCW transmission, with sensitivity analysis performed from 20% to 80%. The third source was from general population case-patients (patient-to-HCW transmission), expressed as the ratio of susceptible HCWs who could be infected by incident case-patients who sought treatment from the healthcare system (H/P). The extent of transmission is dependent on interventions such as barrier precautions (*11*). On the basis of findings from exploratory analysis, increasing the H/P ratio moves the HCW epidemic earlier; at an H/P of 2.08, the HCW epidemic peaks before the start of prophylaxis, negating the outcomes of prophylaxis. Therefore, H/P values >2 do not substantially contribute to the outcomes and study conclusions, and sensitivity analysis was performed for H/P from 0 to 2 (Technical Appendix). Transmission from HCWs to patients was assumed negligible compared with other sources of infection for the general population, and the general population epidemic was independent of transmission dynamics within the HCW population.

Once infected, an HCW would have 4 outcomes based on absenteeism (Figure 1B). Those with asymptomatic infection were assumed to be fit for work. Absenteeism due to symptomatic infection, hospitalization, and death was determined for the different strategies. The study assumed that all HCWs were absent from work while symptomatic and that prophylaxis reduced HCW-to-HCW transmission (*9*). Each scenario was further analyzed on the basis of different R_0;_ the disease’s incubation and infectious periods were kept constant.

### Pandemic Duration and Prophylaxis Initiation

The point of local detection of pandemic influenza depends on various factors and is unknown. Approximately 2,800 cases of influenzalike illness (ILI) occur per day in Singapore (*2*), of which a small fraction is sampled for virologic surveillance (*12*). The base case assumed that the pandemic influenza subtype would be detected when incident symptomatic cases exceeded 10% of baseline ILI rates. The pandemic duration was defined as the period when incident pandemic influenza cases remained above this stated level. Prophylaxis was given to HCWs at the time of disease detection and continued for the planned duration. We conducted sensitivity analysis for starting prophylaxis on introduction of the first case and when incident cases exceeded 1%–100% of the baseline ILI rate.

### Other Input Parameters

The input parameters for analysis (Table 1) were obtained from local sources when available as detailed in a previous study on stockpiling strategies in Singapore (*2*). Other values were obtained from international sources. To account for uncertainties, wide ranges were used for analysis.

**Table 1:** Parameters of neuraminadase inhibitor stockpiling strategies model*

Parameter	Notation†	Minimum‡	Base case‡	Maximum‡	Reference
*Input*					
General population	*N_g_*		4,350,000		(*13*)
Healthcare staff	*N_h_*		20,000		Estimated
ILI rate, per day	ι		2,800		(*2*)
*Transmission dynamics*					
Incubation and latent period, d	α	1.0	2.0	3.0	(*9*,*10*)
Infectious period, d	γ	1.5	4.1	7.0	(*9*,*10*)
Reproductive number	R_0_	1.5	2.5	6.0	(*9*,*14*)
Transmission probability/d	β	0.37	0.61	2.0	Calculated, R/γ
HCW-to-HCW transmission	ω	0.2	0.5	0.8	See text
HCW infections caused by incident cases of clinical influenza (*H/P)*	δ	0		2.0	See text
Detection threshold, proportion of baseline ILI rate	ν	Introduction of 1st case	0.1	1	See text
*Disease severity and antiviral efficacy*					
Hospitalization rate (HCW)/100,000 infected§	η	12.4	88.6	186.7	(*2*)
Length of stay and medical leave if hospitalized, d	φ	9.0	12.0	20.0	(2)
Case-fatality rate (HCW)/100,000 infected§	μ	1.9	20.3	65.1	(*2*)
Proportion of infected persons without prophylaxis who have symptoms	*θ_1_*	0.50	0.67	0.80	(*9*,*15*)
Oseltamivir efficacy for preventing infection in exposed persons	ε_1_	0.28	0.35	0.52	(*9*,*16*,*17*)
Oseltamivir efficacy for preventing disease in infected persons	ε_2_	0.5	0.6	0.9	(*2*,*9*)
Oseltamivir efficacy for preventing transmission of infection by infected persons	ε_3_	0.6	0.8	0.98	(*9*)
Proportion of infected persons receiving oseltamivir prophylaxis who have symptoms	*θ_2_*	0.07	-	0.2	Calculated, *θ_2_ = θ_1_(1−ε_2_)*
Medical leave without treatment, d	σ	2	4	5	(*2*)
Reduction in medical leave with oseltamivir treatment, d	χ	0.1	1.0	2.0	(*2*)
Reduction in hospitalization or case-fatality rate with treatment	ψ	0.4	0.6	0.8	(*2*,*18*)

*HCW, healthcare workers, ILI, influenzalike illness.

†Notations are used in the equations listed in the Appendix.

‡Base case values are given with the minimum and maximum values used in the model where applicable.

§Based on hospitalizations and deaths among those with clinical influenza.

HCWs were assumed to be adults 20–64 years of age with a mix of persons at low and high risk for influenza complications similar to that in the general population. Hospitalization and case-fatality rates were estimated for a pandemic of average severity (*2*). To account for the effect of severe pandemics, a scenario using mortality rates from the 1918 “Spanish flu” (5% average) and correlated hospitalization rates was performed (*19*).

### Outcome Variables and Sensitivity Analysis

Outcome variables from the analyses included pandemic duration, peak staff absenteeism, and days with absenteeism >5%. For parameters relating to disease severity and antiviral efficacy, 1-way sensitivity analysis was performed to determine the effect on outcomes. In addition, Monte Carlo simulation analysis, with 1,000 iterations per scenario, was performed with the range of parameter estimates modeled as triangular distributions. For parameters pertaining to transmission dynamics, separate analyses were performed to determine the effects of variations in HCW-to-HCW and patient-to-HCW transmission. We also tested the outcome effects of assuming different latent and infectious periods. Epidemics with similar R_0_ but different latent and infectious periods have different growth rates. To facilitate comparison between epidemics with different latent and infectious periods, both epidemic growth rates and R_0_ values were presented. The relationship between latent and infectious period, R_0,_ and growth rates was described by Mills et al. (*14*) and elaborated in the Technical Appendix. Finally, the outcomes were determined for the various strategies upon initiation of prophylaxis at different times.

We used Berkeley-Madonna 8.3 software (University of California, Berkeley, CA, USA) to run the model. Details of the equations are shown in the Appendix; additional methods and results are shown in the Technical Appendix.

## Results

**Figure 2:**
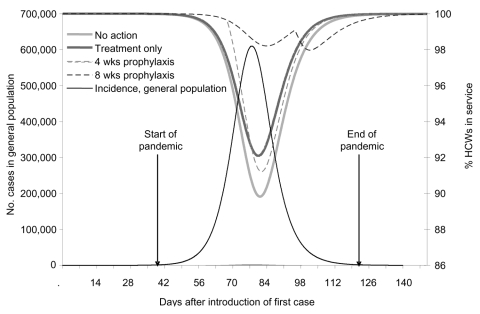
Dynamics of population infections and the effect of different strategies on absenteeism among healthcare workers for a base-case pandemic.

**Figure 3:**
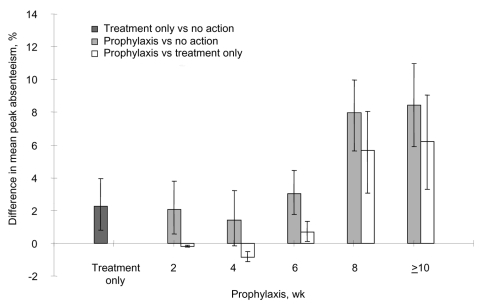
Simulation analysis of the difference in mean peak absenteeism for different strategies in an R_0_ = 2.5 (base-case) pandemic (50th percentile shown in solid bars with the 5th and 95th percentiles shown in error bars).

The epidemic curve for a base-case pandemic with R_0_ of 2.5 had a 12-week duration (Figure 2). When no action was taken, peak HCW absenteeism was ≈10%. Treatment only, using 121,000 doses of oseltamivir, decreased peak absenteeism to 8%. Prophylaxis for 4 weeks required 117,000 treatment doses in addition to 560,000 dedicated prophylaxis doses (equivalent to treatment courses for 1.6% of the general population) and led to higher peak absenteeism than treatment only. Eight weeks of prophylaxis required 52,000 treatment doses in addition to 1.12 million dedicated prophylaxis doses (equivalent to treatment courses for 2.7% of the general population) and reduced peak absenteeism to ≈2%; the peak occurred as a secondary increase after termination of prophylaxis. Discontinuing prophylaxis for clinical infections and redistributing stockpiles to prolong prophylaxis in other HCWs did not provide additional outcome benefits because the doses saved were insignificant; >96% were used during the preplanned duration for the relevant scenarios. From the Monte Carlo simulation of peak absenteeism for different strategies in a pandemic with R_0_ of 2.5, with varying disease severity and antiviral efficacy parameters, 6 weeks of prophylaxis was sufficient under all scenarios to have a net benefit over treatment only (Figure 3).

One-way sensitivity analyses showed that the following input parameters had the most effect on peak absenteeism: “days of medical leave without treatment,” with 15%–96% variation from the baseline outcome, depending on the R_0_ and strategy used; “reduction in medical leave with treatment” with 22%–61% variation; “symptomatic proportion in infected persons without prophylaxis” with 19%–25% variation; and “oseltamivir efficacy in preventing disease in infected persons” with 21%–87% variation. Other input parameters had less effect on the outcome.

Table 2 shows the outcomes for pandemics with different R_0_. If no action was taken for pandemics with R_0_ ≥2, absenteeism exceeded 5% for >15 days. In pandemics with lower R_0_ (≤2), pandemic durations were longer and peak absenteeism did not exceed 10%. Treatment only in these pandemics reduced peak absenteeism by as much as 25% compared with no action. However, prophylaxis of ≤8 weeks did not accrue substantial benefits over treatment only.

**Table 2:** Effects of influenza pandemic prevention strategies on healthcare worker absenteeism

Reproductive no. (R_0_)		Pandemic duration, wk	Peak % absent by strategy (days with >5% absent)					
			No action	Treatment only	2 weeks’ prophylaxis	4 weeks’ prophylaxis	6 weeks’ prophylaxis	8 weeks’ prophylaxis
1.5		24	2.8 (0)	2.1 (0)	2.1 (0)	2.1 (0)	2.2 (0)	2.3 (0)
2		15	6.7 (17.8)	5.1 (5.4)	5.2 (6.5)	5.5 (9.1)	5.9 (11))	4.6 (0)
2.5		12	10.2 (21.1)	7.9 (16)	8.1 (16.2)	8.8 (16.2)	7.2 (10.8)	2 (0)
3		10	13 (20.6)	10.2 (16.6)	10.6 (16.7)	11.4 (15)	4.7 (0)	2.5 (0)
4		8	17.3 (18.7)	13.9 (15.7)	14.6 (15.4)	10.8 (11.1)	3.7 (0)	3.7 (0)
6		6	22.5 (16.5)	18.5 (13.9)	19.7 (12.9)	5.5 (4.1)	5.5 (4.1)	5.5 (4.1)
Pandemic similar to 1918 “Spanish flu”*			20.2 (28.6)	15.1 (18.3)	15.8 (17.9)	11.6 (13)	4.1 (0)	4.1 (0)

*R_0_ =4; mortality rate = 5% (hospitalization set to the ratio of the hospitalization rates to the case-fatality rates in Table 1).

Pandemics with higher R_0_ (≥4) were of shorter durations; peak absenteeism was >20% in some scenarios. Treatment only reduced peak absenteeism by >15%, and 6 weeks of prophylaxis was sufficient to reduce peak absenteeism by >75% over no action. Across all R_0_, insufficient durations of prophylaxis increased peak absenteeism compared with results for treatment only.

During a pandemic similar in severity to the 1918 influenza pandemic, with a 5% mortality rate and R_0_ of 4 (*14*), peak absenteeism reached 20% with no action; hospitalizations and deaths contributed substantially to absenteeism, unlike the situation in less severe pandemics. The 3 strategies—treatment only, 4 weeks of prophylaxis, and 6 weeks of prophylaxis—reduced peak absenteeism by 25%, 43%, and 80%, respectively.

We also tested the adequacy of prophylaxis for a base-case pandemic under different scenarios for HCW-to-HCW and patient-to-HCW transmission. Higher HCW-to-HCW transmission resulted in an increased postprophylaxis epidemic peak. The HCW epidemic coincided with the general population epidemic if the patient-to-HCW infections variable was minimized (H/P = 0). Increasing H/P alone shifted the HCW epidemic such that it preceded the general population epidemic and amplified peak absenteeism by as much as 1.4 for the base case. For the prophylaxis strategies, increasing the patient-to-HCW transmission resulted in the distribution of HCW absenteeism away from the postprophylaxis period into the pre- and intraprophylaxis periods, which resulted in lower peak absenteeism up to a point. For H/P >2.0, peak absenteeism occurred before initiation of prophylaxis, negating the effect of longer durations of prophylaxis. Under all HCW-to-HCW and patient-to-HCW transmission scenarios for a base-case pandemic, 6 weeks of prophylaxis provided equal or superior results to treatment only; 8 weeks of prophylaxis was always superior (Technical Appendix).

**Figure 4:**
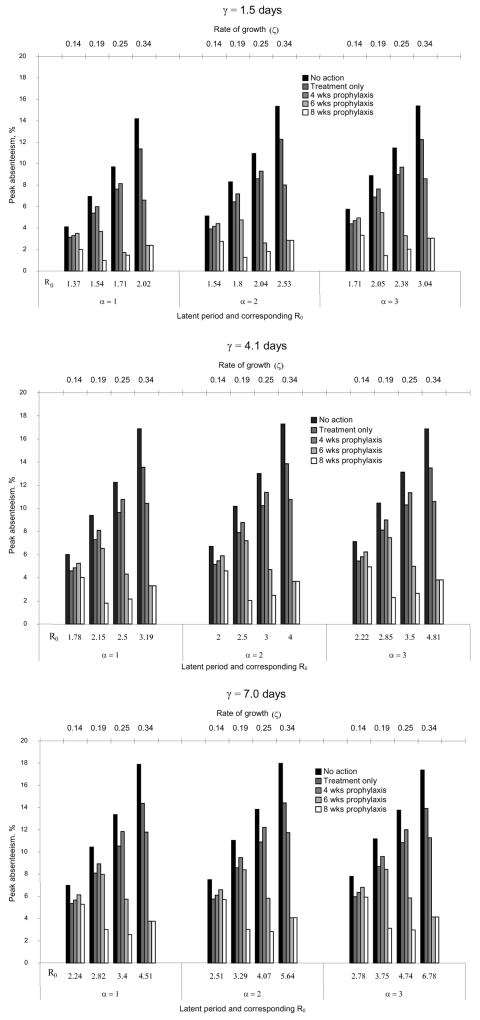
Peak absenteeism with different treatment and prophylaxis strategies varying rates of growth (ζ)*, latent periods (α), and infectious duration (γ).† *ζ is the initial rate of growth of the epidemic curve and is determined by the reproductive potential and the infectious agent’s doubling time (Τ). The latter is related to the rate of growth by the following equation,  
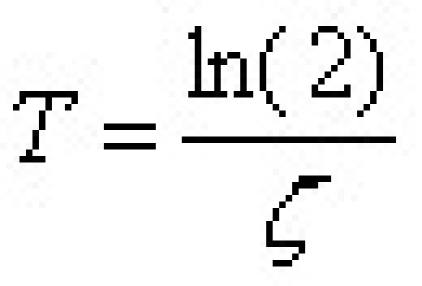
.  †Tx refers to treatment; Rx refers to prophylaxis.

Figure 4 shows the changes in peak absenteeism when latent and infectious periods were varied. For any rate of growth, assuming different latent periods changed peak absenteeism by <1% for most scenarios; assuming longer infectious periods increased peak absenteeism by <3%. However, epidemics with higher growth rates for any latent and infectious periods increased peak absenteeism by >10% when no action was taken. Although changes in the transmission parameters substantially changed peak absenteeism levels for certain scenarios, the overall conclusions remained similar. For epidemics with low peak absenteeism (<10%) and prolonged duration (low growth rate), prophylaxis strategies were less effective than treatment only. In contrast, for epidemics with higher peak absenteeism (>10%) and shorter duration (high growth rate), prophylaxis of >6 weeks was superior to treatment only.

**Figure 5:**
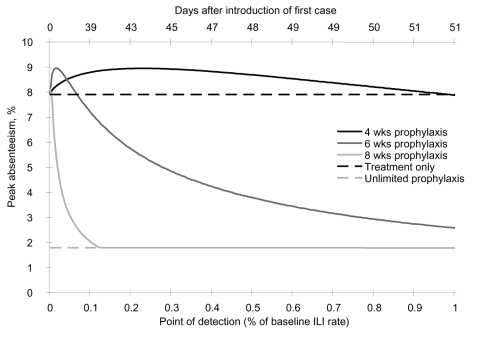
Peak absenteeism observed with different times of initiating prophylaxis, according to point of detection in a base-case pandemic.

Figure 5 shows the adequacy of prophylaxis for a base-case pandemic under different prophylaxis initiation points based on pandemic detection. Earlier detection and prophylaxis initiation resulted in a greater likelihood that shorter durations of prophylaxis would be ineffective. If prophylaxis were initiated on entry of the first pandemic case, 14 weeks of prophylaxis would be required for maximal benefit. Prophylaxis for 6 weeks was more effective than treatment only if it was initiated when incident pandemic cases in the general population exceeded 10% of the ILI rate, whereas 8 weeks of prophylaxis was effective when incident pandemic cases exceeded 1%.

## Discussion

During an influenza pandemic, essential services such as healthcare must be maintained, especially during the pandemic’s peak, when the maximal number of patients require care, and healthcare services can ill afford absenteeism due to infection. Absenteeism may also occur for reasons such as background illnesses and the need to care for ill relatives. During the severe acute respiratory syndrome epidemic in Singapore in 2003, schools were closed for weeks. Although no study documented the resultant workplace absenteeism, parents may have taken time off to care for their children. The New Zealand government has predicted overall absenteeism levels as high as 40% (*20*), and actual pandemic workplace absenteeism levels will likely exceed those shown in this study.

Treatment and timely use of prophylaxis with neuraminidase inhibitors reduce HCW absenteeism compared with no action. As shown in previous studies, treatment provides benefits over no action and should be considered in preparedness plans to reduce illness and death (*2*,*3*,*21*). Using prophylaxis to prevent infection results in a secondary increase in infections after prophylaxis is stopped because susceptible HCWs remain in the general population when transmission occurs. Insufficient durations of prophylaxis thus result in poorer outcomes than treatment only. For prophylaxis strategies to accrue more benefits than treatment only, the prophylaxis duration must be sufficient to cover the pandemic’s peak. Eight weeks of prophylaxis, the maximum safe duration previously studied (*22*), was sufficient to provide a substantial reduction in peak absenteeism under a broad range of assumptions for more severe pandemics where peak absenteeism exceeded 10%. Six weeks of prophylaxis was marginally beneficial, if one assumes that prophylaxis was initiated after incident pandemic cases exceeded 10% of the baseline ILI rate.

An important policy consideration is the timing of prophylaxis initiation. Improved surveillance, critical for early detection, paradoxically increases the likelihood of initiating prophylaxis too early, causing predetermined estimates of stockpile duration to be inaccurate. Many countries have developed comprehensive preparedness plans to reduce a pandemic’s spread. These may prolong the pandemic’s duration within the country, which would compound the issue of stockpile adequacy. If prophylaxis is started prematurely, stockpiles will be exhausted before the delayed waves of the pandemic occur and thus will not reduce absenteeism more than would treatment only. Prophylaxis should not be initiated until a certain point in the epidemic curve, but this may be difficult, given public sentiment and pressure. Further studies are needed to determine the ideal time for prophylaxis initiation and the role of surveillance in evaluating the pandemic phases and projected spread.

The current avian influenza outbreaks have increased fear of an imminent severe pandemic. Pandemics of lesser severity place fewer requirements on essential services. Our study showed that such pandemics also result in lower staff absenteeism rates; treatment and prophylaxis may thus be less critical to service continuity. On the contrary, severe pandemics increase the strain because of the numbers of patients, hospitalizations, and deaths and the reduced response capacity of healthcare services. For pandemics with high mortality rates, high growth rates, or high R_0_, prophylaxis provides greater benefits than it does for pandemics with lower mortality rates, low growth rates, or low R_0_; and the required duration of prophylaxis is shorter.

Our results are subject to several limitations. The true level of transmission in HCWs remains unknown. In a heightened state of alertness, HCWs will be equipped with personal protective equipment, and patient-HCW transmission may be minimized, resulting in lower absenteeism rates (*11*). Another limitation is that effects over the entire HCW population were aggregated. In reality, subsets of HCWs exist with varying levels of exposure. Stochastic variation and nosocomial outbreaks, which were not modeled, may result in higher local absenteeism rates than predicted by this model. Further studies that use individual-based stochastic models may provide improved representation of disease transmission to test other interventions. Studies should also consider modeling the effect of multiple pandemic waves. Finally, the study parameters used were based on historical data; the validity of the projections will depend on how the next pandemic compares with its precedents.

## Conclusion

Countries must consider the effects of an influenza pandemic on essential services. Those planning neuraminidase inhibitor stockpiling for treatment and prophylaxis of essential staff should consider the relatively small quantities required. Treatment and 8 weeks of prophylaxis for HCWs in Singapore costs US $2 million, compared with US $400 million for a similar populationwide stockpile and the ≈US $20 million spent for national stockpiling (*2*). In severe pandemics, when the need for protection is greatest, prophylaxis of short duration has a potential role in mitigating the effects. For prophylaxis strategies to succeed, stockpiles must be adequate and their deployment must be timed to cover the pandemic’s peak. If adequacy and timeliness cannot be achieved, prophylaxis may result in higher absenteeism than treatment only, which makes the latter strategy a more effective option.

## Supplementary Material

Appendix Modified SEIR Model

Technical Appendix Supplementary material including additional methodology, results, and discussion.
