# Supplementary material for: Effectiveness of Neuraminidase Inhibitors for Preventing Staff Absenteeism during Pandemic Influenza
Source: Emerg Infect Dis. 2007 Mar;13(3):449–57. doi: 10.3201/eid1303.060309 (PMC2725890; doi:10.3201/eid1303.060309)
Supplement: Appendix — Modified SEIR Model [file 06-0309_app-s1.pdf]

Appendix

Modified SEIR Model

The model was run across 365 days at time steps of 0.05 days. The equations used in the analysis are shown below; the notations are represented in Table 1.

General Population

For the general population, persons move from the susceptible (S_g) to the exposed (E_g), infected (I_g), and removed (R_g) states as shown in the respective equations below.

$$\frac{d(S_g)}{dt} = -\beta \frac{I_g}{N_g} S_g$$

$$\frac{d(E_g)}{dt} = \beta \frac{I_g}{N_g} S_g - \frac{E_g}{\alpha}$$

$$\frac{d(I_g)}{dt} = \frac{E_g}{\alpha} - \frac{I_g}{\gamma}$$

$$\frac{d(R_g)}{dt} = \frac{I_g}{\gamma}$$

Where *b* is the transmission probability per day from an average infectious person, N_g is the size of the general population, *a* is the incubation period, and γ is the infectious period.

HCW Population

Transmission and disease severity parameters are determined by whether HCWs are given treatment and/or prophylaxis. The use of treatment and prophylaxis is indicated by the variables *i* and *j*, respectively. *i = 0* denotes when treatment is not in use, and *j* = 0 when prophylaxis is not in use, and *i = 1* and *j = 1* denote when treatment and prophylaxis are in use, respectively. The use of prophylaxis is conditional to the pandemic having been detected and the stockpile, *P*, not having been exhausted.

Transmission Dynamics

For the HCW population, persons move through the susceptible (S_h), exposed (E_h), infected (I_h), and removed (R_h), states as shown below:

$$\frac{d(S_h)}{dt} = -(\lambda_h + \lambda_g + \lambda_p)(1 - j\varepsilon_1)S_h$$

$$\frac{d(E_h)}{dt} = (\lambda_h + \lambda_g + \lambda_p)(1 - j\varepsilon_1)S_h - \frac{E_h}{\alpha}$$

$$\frac{d(I_h)}{dt} = \frac{E_h}{\alpha} - \frac{I_h}{\gamma}$$

$$\frac{d(R_h)}{dt} = \frac{I_h}{\gamma}$$

where N_h is the size of the HCW population. j indicates the use of prophylaxis, so that when $j = 1$, HCWs have a reduced susceptibility to infection due to the efficacy of prophylaxis in preventing infection (e_1), λ_h, λ_g and λ_p are the forces of infection acting on HCWs.

λ_h is the force of infection from HCW-to-HCW transmission within the workplace, and is defined as the following:

$$\lambda_h = \omega\beta(1 - j\varepsilon_3)\frac{I_h}{N_h}$$

where ω is the proportional contribution due to HCW-to-HCW transmission to the force of infection, and ε_3 is the efficacy of oseltamivir in reducing infectiousness, which renders a proportion of HCWs on prophylaxis noninfectious when $j = 1$.

λ_g is the force of infection from exposure of HCWs to the general population during the proportion of their time spent outside the workplace. The force of infection is similar to that in the general community, subject to the proportion of time spent outside the workplace (1 – ω). λ_g is thus defined as

$$\lambda_g = (1 - \omega)\beta\frac{I_g}{N_g}$$

λ_p is the additional force of infection from patient-to-HCW transmission due to symptomatic incident patients as they enter the healthcare system with pandemic influenza (occupational hazard). No discrimination between the probability of acquiring infection in the community healthcare or hospital healthcare setting is represented, because the actual probability of transmission in either setting is unknown. Influenza patients are assumed to be distributed randomly among the HCW population and to have an aggregated probability δ of infecting susceptible HCWs with whom they come into contact, regardless of single or multiple contact episodes or duration of contact. The rate at which new symptomatic infections from the general population will present to the healthcare system at any point in time would be $\frac{\theta_1 E_g}{\alpha}$ Therefore, the force of infection for each HCW, λ_p is as follows:

$$\lambda_p = \frac{\delta\theta_1 E_g}{\alpha N_h}$$ where N_h is the number of HCWs under consideration.

We assumed that the small population of infectious HCWs did not affect the transmission dynamics of the disease in the general population.

Absenteeism

HCWs who are exposed will progress from the exposed state (E_h) to the states of asymptomatic infection, clinical infection (C_h), hospitalization (H_h), or death from the disease (D_h). Only the last 3 states contribute to absenteeism according to the respective durations off work as follows:

$$\frac{d(C_h)}{dt} = \theta_{j+1}(1-(1-i\psi)\eta)\frac{E_h}{\alpha} - \frac{C_h}{(\sigma - i\chi)}$$

$$\frac{d(H_h)}{dt} = \theta_{j+1}(1-i\psi)(\eta-\mu)\frac{E_h}{\alpha} - \frac{H_h}{\phi}$$

$$\frac{d(D_h)}{dt} = \theta_{j+1}(1-i\psi)\mu\frac{E_h}{\alpha}$$

where η is the hospitalized proportion, σ is the duration of medical leave in uncomplicated illness, *f* is the duration of hospitalization and subsequent medical leave in complicated illness, and m is the case-fatality proportion. *y* is the reduction in hospitalization or deaths with treatment, and c is the reduction in medical leave with uncomplicated illness with treatment; both these terms are hence only active for values of $i = 1$. q_{j+1} is the symptomatic proportion and hence takes the value of q_1 in the absence of prophylaxis and θ_2 when prophylaxis is used, reflecting the efficacy of prophylaxis in reducing symptomatic disease (e_2).

The number of healthcare staff in operation at any time is hence given as

$$O_h = N_h - C_h - H_h - D_h$$

The proportion absent at any given time is $\frac{O_h}{N_h}$

We ignored the contribution of new recruitments after the start of the epidemic.

Incidence Rates, Start of Pandemic, and Use and Consumption of Prophylaxis Stockpile

The incident number of symptomatic cases of pandemic influenza in the general population, V_g, is given as

$$V_g = \frac{\theta_1 E_g}{\alpha}$$

The pandemic is deemed to start when

$$V_g > \omega t$$

where ι is the baseline ILI rate, and υ is the detection threshold. When $V_E > \upsilon\iota$, then the predetermined stockpile, P, which is expressed as the number of days of prophylaxis stockpiled per HCW, begins to be consumed in strategies that use prophylaxis, i.e.,

$$\frac{d(P)}{dt} = -1$$

In a prophylaxis strategy, $j = 1$ when both conditions, $V_E > \upsilon\iota$ and $P > 0$, are satisfied; otherwise, $j = 0$.
