# Supplementary material for: Effectiveness of Neuraminidase Inhibitors for Preventing Staff Absenteeism during Pandemic Influenza
Source: Emerg Infect Dis. 2007 Mar;13(3):449–57. doi: 10.3201/eid1303.060309 (PMC2725890; doi:10.3201/eid1303.060309)

# Technical Appendix

## Supplementary material including additional methodology, results, and discussion.

## Additional Methods

Treatment and prophylaxis stockpiles

Under the treatment only strategy, we considered the possibility that the treatment stockpiles may be limited. We analyzed the effect of varying the percentage of infected receiving treatment, on the outcome of peak absenteeism; for different $R_0$. Under the prophylaxis strategies, we assumed that treatment stockpiles would be large enough to ensure sufficient treatment doses are available above those planned for use as prophylaxis.

We also explored the scenario that although the prophylaxis stockpiles are fixed at a certain quantity, a proportion of HCWs may develop clinical illness either before the start of, or during, prophylaxis. If these clinically infected HCWs can be identified as pandemic influenza infections, they would not need to continue receiving prophylaxis. The result is that some prophylaxis doses may be saved; and these saved doses may potentially be redistributed to the other non-clinically infected HCWs, prolonging prophylaxis beyond the planned duration. For example, if we originally stockpiled for 6 weeks of prophylaxis, and some HCWs could stop prophylaxis because they were clinically infected prior to the start of or during prophylaxis, then some doses could be saved and redistributed to other HCWs. This prolongs the duration of prophylaxis beyond the original 6 weeks. We have performed analyses to explore this scenario, although this is only possible if tests can promptly confirm individual infection and logistics networks allow for prompt redistribution. To address this issue from another angle, we explored the number of prophylaxis doses used at the end of the planned duration of prophylaxis for the various scenarios (based on $R_0$) if those who are clinically infected can be identified and prophylaxis stopped.

The total amount of oseltamivir used was also analyzed under the assumption that all HCWs consumed prophylaxis for the pre-planned duration, and ignoring the effect of the handful of deaths during prophylaxis and the few doses that may be saved when clinically infected HCWs on prophylaxis receive treatment doses drawn from the treatment stockpile.

Transmission dynamics

Transmission dynamics plays an important role in determining the growth of the epidemic and the shape of the epidemic curve, which in turn determines the overall epidemic duration and peak absenteeism. Similar to another modeling study, we assumed onset of symptoms coincided with the onset of infectiousness i.e. that the incubation period coincided with the latent period (1). The actual difference in timing and duration is probably less than a day for influenza since symptoms start on the same day as detectable viral shedding (2), and we hence assumed the same value (and corresponding symbol) to describe the incubation and latent periods in our study.

For our base case, we assumed a latent/incubation period of 2 days and an infectious period of 4.1 days, similar to base case values used by Mills et al in estimating $R_0$ from the 1918 pandemic (3). We then generated a set of epidemics with a range of growth rates by changing $R_0$ based on the above latent and infectious periods.

Outcome variables and sensitivity analysis

At lower $R_0$ of 2 or less, the impact of mis-timed prophylaxis is less of an issue, since the overall and peak absenteeism is low. At higher $R_0$ of more than 4, the epidemic progresses so quickly (about 6 weeks duration) that prophylaxis stockpiles will be sufficient to achieve their intended effect. The key scenarios of concern are those with $R_0$ between 2.5 to 4, as mis-timed prophylaxis can substantially exacerbate the effects of the outcomes. Under these scenarios, 4 to 8 weeks of prophylaxis can either be subtantially more or less effective in reducing peak absenteeism compared to treatment only. Most of our sensitivity analyses were hence focussed on these combinations of scenarios.

Outcome variables from the analyses included pandemic duration, peak staff absenteeism, and days with absenteeism above 5%. We have focused our attention on peak staff absenteeism in the sensitivity analyses as a marker for comparison of the pandemic’s impact on business continuity, as this will influence other outcomes. The number of days with absenteeism above 5% varies with peak absenteeism and pandemic duration (depending on the $R_0$), and does not provide for independent comparison across scenarios.

For parameters relating to disease severity and antiviral efficacy (previously studied parameters), one-way sensitivity analysis was performed to determine the impact on the outcomes. We performed separate one-way sensitivity analysis with different combinations of $R_0$ and management strategies (no action, treatment only, and prophylaxis). This is because each combination of $R_0$ and management strategy affects the outcomes on varying the input parameters. Certain input parameters such as efficacy of prophylaxis and effectiveness of treatment are not applicable to the strategies of treatment and no action, and were therefore excluded during analyses for the respective strategies. To facilitate interpretation on the effect of prophylaxis, we present results for sustained prophylaxis for the entire pandemic duration. Hospitalization and case fatality rates were scaled together based on their distributions, with the upper and lower limits fixed for both variables in a distribution centered on the mean. This is because hospitalization and case fatality rates are likely correlated during a pandemic (4).

In addition, Monte Carlo simulation analyses, with 1,000 iterations per scenario, were performed with the range of disease severity and antiviral efficacy parameter estimates modeled as triangular distributions. The result for the base case scenario has been shown in Figure 3 of the main manuscript, and we present the median, 5$^{th,}$, and 95$^{th}$ percentiles based on the various $R_0$ and strategies.

Parameters pertaining to transmission dynamics were analyzed separately because these values are future predictions whose distributions cannot be predicted by exisiting studies.

Sensitivity analyses based on multiple scenarios were also performed to determine if variation in HCW-to-HCW and patient-to-HCW transmission affected the outcomes. We explored one-way sensitivity analyses on these parameters for the outcomes of peak absenteeism; and the timing of peak absenteeism from introduction of the first case in the general population. We then explored the combined effect of varying both patient-to-HCW and HCW-to-HCW transmission parameters simultaneously in two-way sensitivity analyses.

To address the concern about how the different combinations of latent and infectious periods may affect the results, we conducted sensitivity analysis in which different latent and infectious periods were used. However, the growth rate of an epidemic is determined both by the reproductive potential as well as its generation time of the infectious agent (i.e. the time it takes to produce the sucessive generation of cases); for example, an epidemic caused by an infectious agent with an $R_0$ of 2 but a generation time of 3 days would grow at the same rate as an epidemic with an $R_0$ of 4 but a generation time of 6 days. To account for this, we defined a set of epidemics based on their growth rates, $\zeta$, corresponding to $R_0$ = 2.0 to 4.0 with a latent period, $\alpha$, of 2 days and an infectious period, $\gamma$, of 4.1 days. We then recalculated the corresponding $R_0$ for different parameter choices of $\alpha$ amd $\gamma$ based on the equation given by Mills et al (3). The equation is reproduced below, using our chosen notation for growth rates, and latent and infectious periods:

$$R_0 = 1 + \zeta.(\alpha + \gamma) + \alpha.\gamma.\zeta^2$$

We modelled a broad range for latent and infectious periods, from $\alpha = 1$ to $\alpha = 3$ and from $\gamma = 1.5$ to $\gamma = 7$ (Table 1 of the main manuscript).

## Additional Results

Figure A1 explores the sufficiency of treatment stockpiles for different $R_0$. The outcomes of varying the percentage of infected individuals receiving treatment (due to limited stockpiles) lie progressively between the outcomes under no action and full treatment of all infected HCWs.

Table A1 compares peak absenteeism for HCW prophylaxis with re-distribution and without re-distribution (fixed pre-planned duration) of the prophylaxis doses. Redistribution of prophylaxis doses had none or only a marginal effect (in a few scenarios) on reducing peak absenteeism.

Table A2 shows the number of prophylaxis doses used at the end of the planned duration of prophylaxis for the various scenarios (based on $R_0$). For lower $R_0$ (≤2.5) or shorter pre-determined durations of prophylaxis (≤4 weeks), more than 90% of prophylaxis stocks were utilised by the end of the pre-determined duration of prophylaxis. For higher $R_0$ (≥3) or longer pre-determined durations of prophylaxis (>8 weeks), less stocks were used but re-distribution of prophylaxis did not reduce peak absenteesim (Table A1) since pre-planned durations would already have been adequate. For the important scenarios (scenarios where an incremental increase in prophylaxis duration resulted in a sharp decrease in peak absenteeism as shown in Table A1), prophylaxis doses utilized remained above 93%.

Table A3 shows the treatment and prophylaxis doses required under the various strategies for the base case scenario ($R_0$ =2.5). Prophylaxis doses constitute the overwhelming majority of the doses required for prophylaxis strategies of 4 weeks and above. The number of treatment doses required decreased for longer durations of prophylaxis, but the number of treatment doses saved under the different prophylaxis strategies is relatively negligible considering the number of prophylaxis doses required.

<u>Disease severity and anti-viral efficacy parameters</u>

For the following results, the scenarios with values of $R_0$ from 2.5 to 4.0, and the most viable strategies of 4 to 8 weeks of prophylaxis should be focused on, because of their substantial impact on the outcome.

Figures A2 to A4 show the results of one-way sensitivity analyses with different combinations of $R_0$ and management strategies. Regardless of the values of $R_0$, for a given strategy, the outcomes are most sensitive to the same parameters.

For the strategy of no action, “days of medical leave without treatment” and “symptomatic proportion in infected persons without prophylaxis” had a substantial effect on the outcomes. “Days of medical leave without treatment” had 15% to 49% variation from the baseline outcome depending on the $R_0$; while “symptomatic proportion in infected persons without prophylaxis” had 19% to 25% variation. The outcomes were insensitive to hospitalization, case-fatality and the length of hospitalization in symptomatic infections.

The treatment only strategies were sensitive to the “reduction in medical leave with oseltamivir treatment”, in addition to “days of medical leave without treatment” and “symptomatic proportion in infected persons without prophylaxis”. “Days of medical leave without treatment” had 20% to 96% variation from the baseline outcome depending on the $R_0$; “reduction in medical leave with treatment” had 22% to 60% variation; and “symptomatic proportion in infected persons without prophylaxis” had 19% to 25% variation. The outcomes were insensitive to the other input parameters.

Prophylaxis strategies were also sensitive to the efficacy of anti-virals when used as prophylaxis, such as “oseltamivir efficacy in preventing infection in exposed persons”, “oseltamivir efficacy in preventing disease in infected persons”, “oseltamivir efficacy in preventing transmission of infection by infected persons”; in addition to the factors for treatment only. “Oseltamivir efficacy in preventing disease in infected persons” had 21% to 87% variation from the baseline outcome depending on the $R_0$; “oseltamivir efficacy in preventing infection in exposed persons” had 5% to 25% variation; “oseltamivir efficacy in preventing transmission of infection by infected persons” had 5% to 8% variation; “days of medical leave without treatment” had 25% to 75% variation; “reduction in medical leave with treatment” had 23% to 61% variation; and “symptomatic proportion in infected persons without prophylaxis” had 19% to 25% variation. The outcomes were insensitive to the other input parameters.

Table A4 gives the multi-way sensitivity analysis using Monte-Carlo simulation (1,000 iterations) for disease severity and anti-viral efficacy parameters. For $R_0 \geq 2.5$, 8

weeks of prophylaxis provided results that were sufficiently close to providing prophylaxis throughout the entire pandemic. For lower $R_0$ (≤2), prophylaxis for 6 to 8 weeks provided better outcomes compared to no action but not necessarily to treatment only. Outcomes for no action and treatment only were subject to a greater spread of uncertainty than those with adequate prophylaxis. For the base-case scenario ($R_0$=2.5, Figure 3, main manuscript), 6 weeks of prophylaxis had a marginal advantage over treatment only, while 8 weeks or more had a clear advantage over treatment.

Other parameters pertaining to transmission dynamics

Tables A5 and A6 show that the transmission dynamics parameters affect both the intensity of transmission within the HCW population, as well as the timing of the HCW epidemic.

From a baseline of no patient-to-HCW transmission, even a small increment of patient-to-HCW transmission had the potential to increase peak absenteeism in HCWs (Table A5); the effect, however, saturated at higher values of H/P. With regards to epidemic timing, when patient-to-HCW transmission was minimized (H/P = 0), the HCW epidemic peaked at the same time as the peak in the general population. Increasing the H/P ratio shifted the HCW epidemic forward, such that it precedes that in the general population. At extreme values of H/P, the HCW epidemic peaked before the start of HCW prophylaxis. This occurred at about H/P = 2.08 for base case parameters. Therefore, for the subsequent analyses, we used values for H/P up to 2.

As shown in Table A6, changing the extent of transmission attributable to HCW-to-HCW contact had minimal effect on both the peak absenteeism and the timing of the HCW epidemic.

Figures A5 to A10 show the combined effect of varying both patient-to-HCW and HCW-to-HCW transmission parameters simultaneously in two-way sensitivity analyses. For all relevant combinations of patient-to-HCW and HCW-to-HCW transmission shown with $R_0$=2.5, 6 weeks of prophylaxis was sufficient to be at least marginally superior to treatment only, while 8 weeks of prophylaxis was clearly superior to the treatment only strategy. For pandemics of shorter durations (either in the entire population with higher $R_0$; or within the HCW population with an increased H/P ratio), shorter durations of prophylaxis are superior to treatment only – the reduction of peak absenteeism for 4 weeks of prophylaxis were as effective as 8 weeks prophylaxis. For pandemics of longer durations (lower $R_0$ or decreased H/P ratio), prophylaxis is inferior to treatment only. At $R_0$ of 1.5 and at lower H/P, even 8 weeks of prophylaxis is insufficient. However, for these longer duration pandemics, overall peak absenteeism is already low.

Latent and infectious periods

Figure 4 in the main manuscript shows the peak absenteeism with different treatment and prophylaxis strategies varying rates of growth ($\zeta$), latent periods ($\alpha$), and infectious durations ($\gamma$). The centre set of figures in A12 with $\alpha = 2$ and $\gamma = 4.1$ was with our base case parameters. At low growth rates, although situations of inadequate prophylaxis are more likely, peak absenteeism is low (<10%) regardless of the

strategy chosen, and is relatively insensitive to the choice of prophylaxis duration. At higher growth rates where peak absenteeism is >10%, 6 weeks of prophylaxis is equal or superior to treatment alone, and 8 weeks is always substantially superior.

Discussion

From Figure A1, optimal results for the treatment only strategy are possible even without stockpiling of treatment doses for 100% of HCWs for a few reasons. Firstly, a proportion of HCWs remain uninfected during the pandemic; this proportion is dependent on the $R_0$, and decreases with increasing $R_0$ because of the larger number of secondary infections. In addition, 33% of infected HCWs would be asymptomatic (base-case assumptions), and will not require treatment. Finally, to achieve suppression of peak absenteeism, the treatment stockpile only needs to cover slightly past the epidemic's peak; further treatment during the tail end of the epidemic will not have any effect on the peak. For example, in a base case pandemic with $R_0$=2.5, stockpiling for about 40% of HCWs would be sufficient to achieve optimal results (Figure A1).

However, pandemic preparedness plans should guard against all possibilities of spread. This would include the possibility of a 2nd or 3rd wave, the absence of effective vaccines, and increased infection rates for high-risk sub-populations such as HCWs. We assumed that sufficient treatment doses are available as planned in current prophylaxis strategies, because prophylaxis is always over and above stocks available for treatment. This may necessitate having 100% treatment coverage for all HCWs.

From the results, it is apparent that prophylaxis must cover the pandemic's peak to achieve a reduction of peak absenteeism over the treatment only strategy. As pandemics with higher $R_0$ (≥4) are 8 weeks or less in duration, stockpiles of 8 weeks would cover the entire pandemic duration. Additional stockpiles in such situations will not accrue additional benefits but only increase costs. To protect HCWs in the worst-case scenarios such as pandemics with high $R_0$, fast spread, and high peak absenteeism; prophylaxis strategies for 6 to 8 weeks will be effective. This shields HCWs from the majority of infections occuring in the general population, leaving them to provide critical healthcare services during the pandemic's peak.

Under all circumstances, redistributing prophylaxis to extend the prophylaxis duration beyond the pre-determined duration does not have a substantial effect on peak absenteesim (Tables A1 and A2). This is because the utilization of prophylaxis doses is more than 93% for the important scenarios as mentioned above. For the scenarios where utilization falls below 90%, the majority of infections have taken place before the end of the pre-determined prophylaxis duration. In these situations, the redistribution of prophylaxis doses does not have substantial impact on absenteeism because the pandemic's peak has passed.

Current pandemic plans call for the distribution and consumption of prophylaxis for the specified duration because clinical influenza infection cannot be easily determined given the presence of other influenza-like illnesses, even with laboratory tests which will require time to develop and distribute. The only savings in prophylactic doses may be from the very small number of HCW deaths during prophylaxis, and from the fact that for every HCW developing clinical illness while on prophylaxis, 5 doses will

be saved from the prophylaxis stockpile if we draw the entire treatment course from the separate treatment stockpile. The duration of prophylaxis for all HCWs was therefore used to represent the strategies, as per current pandemic preparedness protocols, as it presents the most conservative scenario where the stockpiles are maximally utilized (although we have shown that either method of utilization results in similar conclusions).

From the one-way sensitivity analyses in Figures A2 and A4, the input parameter of “reduction in medical leave with treatment” and the parameters pertaining to the effects of prophylaxis all had substantial impact on the outcome. This shows that the outcome of peak absenteeism was sensitive to the treatment and prophylaxis strategies being considered in this study.

As shown in Table A1 and A4, treatment only was always superior to no action and should always be considered in preparedness plans. However, insufficient durations of prophylaxis can be detrimental compared to treatment only, depending on the assumptions about transmission dynamics, disease severity, and antiviral efficacy. Low $R_0$ pandemics with long durations tend to render prophylaxis insufficient. However, in these pandemics, the slow pick-up in the epidemic curve and relatively low peak absenteeism may allow policy makers to choose the appropriate strategy based on initial surveillance data.

From Table A6, changing the proportion of transmission attributable to HCW-to-HCW spread had a minimal effect on both peak absenteeism and timing of the HCW epidemic. This is because disease transmission among HCWs is dependent on HCW-to-HCW spread as well as acquisition of disease from the general population. These two modes of spread are correlated (Appendix 1) – increasing one proportion decreases the other, possibly negating the effects of the changes. The additional increase in peak absenteeism resides on patient-to-HCW spread, which is in turn dependent on the amount of protection provided to HCWs. Infection control and personal protective equipment may thus be important aspects of HCW protection during a pandemic.

Figures A5 to A10 reinforce the fact that for pandemics of shorter durations, shorter durations of prophylaxis are effective because they are sufficient to cover most of the pandemic’s duration. It is during these pandemics (shorter duration and high peak absenteeism) that the impact will be greatest and where prophylaxis strategies will be effective.

Finally, because there have been different estimates of latent and infectious periods, we determined whether our conclusions would have been affected had different latent and infectious periods been assumed while fixing the growth rates of the epidemics. We see that, even for a broad range of epidemic scenarios, even very extreme choices of values for the latent period and infectious period would have little impact on the conclusions (Figure 4 of the main manuscript).

Policy Implications

Policy makers must consider stockpiling sufficient anti-virals to treat clinically infected HCWs. In addition, policy makers should consider prophylaxis from a risk

management perspective. Severe pandemics increase the strain on HCWs due to the numbers of patients and hospitalizations, and the reduced response capacity of healthcare services. Policies should therefore consider protection against high impact pandemics of short duration, high morbidity and mortality, and high peak absenteeism. In these pandemics, prophylaxis durations of 6 to 8 weeks will be effective across a range of scenarios, and have been shown in studies to be safe (5). As the amount of prophylaxis available for critical workers is relatively small compared to strategies for the entire country – such an investment may be cost-beneficial since critical functions cannot be sacrificed. While we prepare for worst-case scenarios, the actual pandemic may be prolonged and of lower impact. Pandemics of lesser severity will probably place fewer requirements on essential services, and this study showed that such pandemics also result in lower absenteeism rates – treatment and prophylaxis is less critical to service continuity. For such pandemics, policy makers will have sufficient time to reconsider their options during the pandemic itself.

Policy makers must also consider additional preventive measures in addition to anti-viral drugs. Public health and infection control measures must be emphasized together with anti-viral use, and not superseded by treatment or prophylaxis strategies.

Finally, surveillance networks are important to ensure that the appropriate strategy is adopted based on the projected epidemic curve during the early pandemic phases. Policy makers must be informed that untimely prophylaxis is detrimental to the outcome. Prophylaxis initiation should be held back until a certain point in the epidemic curve where prophylaxis has substantial impact and covers the pandemic's peak, although this may be difficult given public sentiment and pressure. Premature initiation may render prophylaxis less or ineffective. Information acquired from surveillance should influence policy decision appropriately, and further studies are needed to determine the ideal time for prophylaxis initiation and the role of surveillance in evaluating the pandemic phases and projected spread. If prophylaxis initiation is premature, treatment only may be the better option to reduce absenteeism.

References

1. Longini IM Jr, Halloran ME, Nizam A, Yang Y. Containing pandemic influenza with antiviral agents. Am J Epidemiol. 2004; 159:623-33.
2. Hayden FG, Fritz R, Lobo MC, Alvord W, Strober W, Straus SE. Local and systemic cytokine responses during experimental human influenza A virus infection. Relation to symptom formation and host defense. J Clin Invest. 1998; 101:643-9.
3. Mills CE, Robins JM, Lipsitch M. Transmissibility of 1918 pandemic influenza. Nature. 2004; 432:904-6.
4. Lee VJ, Phua KH, Chen MI, Chow A, Ma S, Goh KT, and Leo YS. Economics of neuraminidase inhibitor stockpiling for pandemic influenza, Singapore. Emerg Infect Dis. 2006; 12:95-102.
5. Chik KW, Li CK, Chan PKS, Shing MMK, Lee V, Tam JSL, et al. Oseltamivir prophylaxis during the influenza season in a paediatric cancer centre: prospective observational study. Hong Kong Med J 2004; 10:103-6.

Table A1. Peak absenteeism by reproductive number and anti-viral strategy, with and without redistribution of prophylaxis doses

| Reproductive number, $R_0$ | Pandemic duration in weeks | Peak % absent by strategy without redistribution (peak % with redistribution, where applicable) | | | | | | | | |
|---|---|---|---|---|---|---|---|---|---|---|
| | | No action | Treatment only | Planned duration of prophylaxis, in weeks | | | | | | |
| | | | | 2 | 4 | 6 | 8 | 10 | 12 | 14 |
| 1.5 | 24 | 2.8 | 2.1 | 2.1 (2.1) | 2.1 (2.1) | 2.2 (2.2) | 2.3 (2.3) | 2.4 (2.4) | 2.1 (2.1) | 1.4 (1.4) |
| 2 | 15 | 6.7 | 5.1 | 5.2 (5.2) | 5.5 (5.5) | 5.9 (5.9) | 4.6 (4.5) | 1.8 (1.5) | 1.1 (1.1) | 1.1 (1.1) |
| 2.5 | 12 | 10.2 | 7.9 | 8.1 (8.1) | 8.8 (8.8) | 7.2 (7.0) | 2.0 (1.8) | 1.8 (1.8) | 1.8 (1.8) | 1.8 (1.8) |
| 3 | 10 | 13 | 10.2 | 10.6 (10.6) | 11.4 (11.4) | 4.7 (3.9) | 2.5 (2.5) | 2.5 (2.5) | 2.5 (2.5) | 2.5 (2.5) |
| 4 | 8 | 17.3 | 13.9 | 14.6 (14.6) | 10.8 (10.1) | 3.7 (3.7) | 3.7 (3.7) | 3.7 (3.7) | 3.7 (3.7) | 3.7 (3.7) |
| 6 | 6 | 22.5 | 18.5 | 19.7 (19.7) | 5.5 (5.5) | 5.5 (5.5) | 5.5 (5.5) | 5.5 (5.5) | 5.5 (5.5) | 5.5 (5.5) |
| Pandemic similar to 1918 "Spanish Flu"* | | 20.2 | 15.1 | 15.8 (15.8) | 11.6 (11.0) | 4.1 (4.1) | 4.1 (4.1) | 4.1 (4.1) | 4.1 (4.1) | 4.1 (4.1) |

* $R_0 = 4$, mortality = 5%, (hospitalization set to the ratio of the hospitalization rates to the case fatality rates in Table 1)

Table A2. Prophylaxis doses utilized at the end of the pre-determined prophylaxis period, under the assumption that prophylaxis can be redistributed.

| Reproductive number ($R_0$) | Number of prophylaxis doses used at the end of the pre-determined prophylaxis period (% of total prophylaxis stockpile), by weeks of prophylaxis | | | | | | |
|---|---|---|---|---|---|---|---|
| | 2 weeks | 4 weeks | 6 weeks | 8 weeks | 10 weeks | 12 weeks | 14 weeks |
| 1.5 | 279,722 (99.9%) | 559,834 (100%) | 839,146 (99.9%) | 1,117,660 (99.8%) | 1394290 (99.6%) | 1666850 (99.2%) | 1935180 (98.7%) |
| 2 | 279,823 (99.9%) | 559,817 (100%) | 837,308 (99.7%) | 1,106,720 (98.8%) | 1363390 (97.4%) | 1612570 (96%) | 1860210 (94.9%) |
| 2.5 | 279,843 (99.9%) | 559,258 (99.9%) | 829,496 (98.7%) | 1,079,280 (96.4%) | 1319340 (94.2%) | 1557250 (92.7%) | 1795360 (91.6%) |
| 3 | 279,837 (99.9%) | 557,752 (99.6%) | 814,639 (97%) | 1,050,350 (93.8%) | 1282130 (91.6%) | 1513100 (90.1%) | 1744450 (89%) |
| 4 | 279,769 (99.9%) | 549,723 (98.2%) | 782,356 (93.1%) | 1,005,170 (89.7%) | 1252010 (89.4%) | 1477770 (88%) | 1703940 (86.9%) |
| 6 | 279,262 (99.7%) | 524,068 (93.6%) | 738,258 (87.9%) | 950,818 (84.9%) | 1227310 (87.7%) | 1449020 (86.3%) | 1671110 (85.3%) |

Table A3. Treatment and prophylaxis doses required for the base case scenario under the assumption that prophylaxis is consumed by all HCWs for the pre-planned duration. Equivalent treatment doses for the general population are shown.

| Strategy | | Treatment doses | Prophylaxis doses | *Equivalent treatment doses for the general population (%) |
|---|---|---|---|---|
| Treatment only | | 121,158 | 0 | 0.28 |
| Prophylaxis | 2 weeks | 120,889 | 280,000 | 0.92 |
| | 4 weeks | 117,337 | 560,000 | 1.56 |
| | 6 weeks | 91,330 | 840,000 | 2.14 |
| | 8 weeks | 52,098 | 1,120,000 | 2.69 |
| | 10 weeks | 35,383 | 1,400,000 | 3.30 |
| | 12 weeks | 32,034 | 1,680,000 | 3.94 |
| | 14 weeks | 31,559 | 1,960,000 | 4.58 |

* includes sum of treatment and prophylaxis doses used for HCWs

Table A4: Multi-way sensitivity analysis for peak HCW absenteeism under different strategies and values of $R_0$

| Reproductive number, $R_0$ | Peak absenteeism, Median % ($5^{th}$, $95^{th}$ percentile) | | | | | |
|---|---|---|---|---|---|---|
| | No action | Treatment only | Planned duration of prophylaxis, in weeks | | | |
| | | | 4 | 6 | 8 | Prophylaxis throughout* |
| 1.5 | 2.8 (1.9,3.6) | 2.1 (1.1,3) | 2.2 (1.1,3.1) | 2.2 (1.1,3.1) | 2.3 (1.2,3.3) | 0.3 (0.1,0.6) |
| 2 | 6.7 (4.5,8.6) | 5.1 (2.7,7.3) | 5.6 (2.8,7.8) | 6.0 (3.2,8.3) | 4.6 (2.5,6.6) | 0.9 (0.4,1.6) |
| 2.5 | 10.3 (7,12.8) | 8.0 (4.4,11.1) | 9.0 (4.8,12.0) | 7.3 (4.0,9.9) | 2.1 (1.1,3.1) | 1.6 (0.7,2.6) |
| 3 | 13.2 (8.8,16.4) | 10.3 (5.2,14) | 11.5 (6.6,15.5) | 4.7 (2.6,6.7) | 2.2 (0.9,3.6) | 2.2 (0.9,3.6) |
| 4 | 17.4 (12.3,21.5) | 13.9 (7.6,18.7) | 10.9 (6.3,14.5) | 3.3 (1.5,5.4) | 3.3 (1.3,5.3) | 3.3 (1.3,5.3) |
| 6 | 22.5 (16.3,27.6) | 18.8 (10.8,24.3) | 5.0 (2.5,7.8) | 4.9 (2.1,7.9) | 4.9 (2.0,8.0) | 4.9 (2.1,7.9) |

*Assumes prophylaxis is sufficient to cover entire pandemic duration

Table A5: Effect of changing $R_0$ and patient-to-HCW transmission (H/P ratio) on peak absenteeism and timing of peak absenteeism

| $R_0$ | 1.5 | | 2.0 | | 2.5 | |
|---|---|---|---|---|---|---|
| Patient-to-HCW transmission (H/P ratio) | Peak absenteeism without treatment, HCWs (%) | Timing of peak absenteeism, HCWs* | Peak absenteeism without treatment, HCWs (%) | Timing of peak absenteeism, HCWs* | Peak absenteeism without treatment, HCWs (%) | Timing of peak absenteeism, HCWs* |
| 0 | 2.8 | 190.1 | 6.7 | 110.3 | 10.2 | 81.2 |
| 0.01 | 4.3 | 177.9 | 8.6 | 102.7 | 12.1 | 75.7 |
| 0.1 | 5.7 | 154.4 | 10.4 | 88.5 | 14.0 | 65.9 |
| 1 | 6.0 | 124.5 | 10.8 | 72.9 | 14.3 | 54.3 |
| 10 | 6.1 | 93.7 | 10.8 | 56.3 | 14.4 | 42.5 |
| Timing of peak prevalence, general population* | 190.1 | | 110.3 | | 81.2 | |
| $R_0$ | 3.0 | | 4.0 | | 6.0 | |
| Patient-to-HCW transmission (H/P ratio) | Peak absenteeism without treatment, HCWs (%) | Timing of peak absenteeism, HCWs* | Peak absenteeism without treatment, HCWs (%) | Timing of peak absenteeism, HCWs* | Peak absenteeism without treatment, HCWs (%) | Timing of peak absenteeism, HCWs* |
| 0 | 13.0 | 65.8 | 17.3 | 49.5 | 22.5 | 35.3 |
| 0.01 | 14.8 | 61.4 | 18.8 | 46.4 | 23.4 | 33.3 |
| 0.1 | 16.7 | 53.6 | 20.6 | 40.6 | 25.0 | 29.3 |
| 1 | 17.1 | 44.4 | 21.0 | 34.0 | 25.4 | 24.7 |
| 10 | 17.2 | 35.1 | 21.1 | 27.2 | 25.5 | 20.2 |
| Timing of peak prevalence, general population* | 65.8 | | 49.5 | | 35.3 | |

*Time in days from introduction of first infectious case

Table A6: Effect of changing $R_0$ and HCW-to-HCW transmission ($\omega$) on peak absenteeism and timing of peak absenteeism

| $R_0$ | 1.5 | | 2.0 | | 2.5 | |
|---|---|---|---|---|---|---|
| HCW-to-HCW transmission (ω) | Peak absenteeism without treatment, HCWs (%) | Timing of peak absenteeism, HCWs* | Peak absenteeism without treatment, HCWs (%) | Timing of peak absenteeism, HCWs* | Peak absenteeism without treatment, HCWs (%) | Timing of peak absenteeism, HCWs* |
| 0.2 | 2.8 | 190.0 | 6.7 | 110.2 | 10.2 | 81.2 |
| 0.5 | 2.8 | 190.0 | 6.7 | 110.2 | 10.2 | 81.2 |
| 0.8 | 2.8 | 190.0 | 6.7 | 110.3 | 10.2 | 81.3 |
| Timing of peak prevalence, general population* | 190.1 | | 110.3 | | 81.2 | |
| $R_0$ | 3.0 | | 4.0 | | 6.0 | |
| HCW-to-HCW transmission (ω) | Peak absenteeism without treatment, HCWs (%) | Timing of peak absenteeism, HCWs* | Peak absenteeism without treatment, HCWs (%) | Timing of peak absenteeism, HCWs* | Peak absenteeism without treatment, HCWs (%) | Timing of peak absenteeism, HCWs* |
| 0.2 | 13.0 | 65.8 | 17.3 | 49.5 | 22.5 | 35.3 |
| 0.5 | 13.0 | 65.8 | 17.3 | 49.5 | 22.5 | 35.3 |
| 0.8 | 13.1 | 65.9 | 17.4 | 49.6 | 22.6 | 35.4 |
| Timing of peak prevalence, general population* | 65.8 | | 49.5 | | 35.3 | |

*Time in days from introduction of first infectious case

Figure A1. Peak absenteeism for different treatment stockpile sizes, under different $R_0$

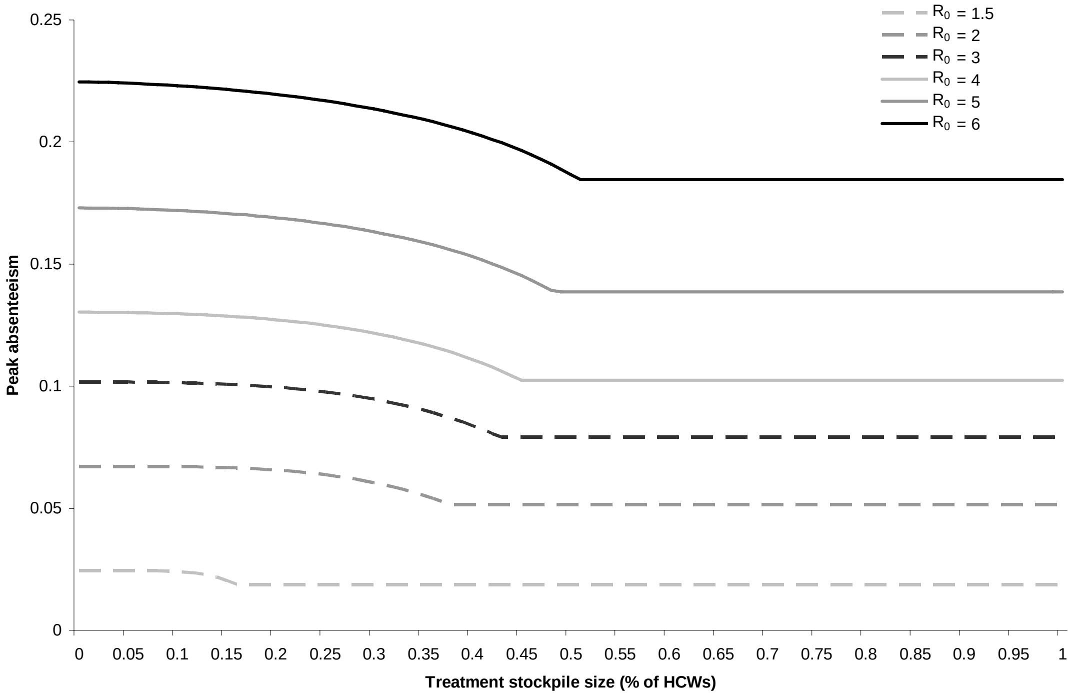

Figure A2. One-way sensitivity analysis for the strategy of no action, by $R_0$.

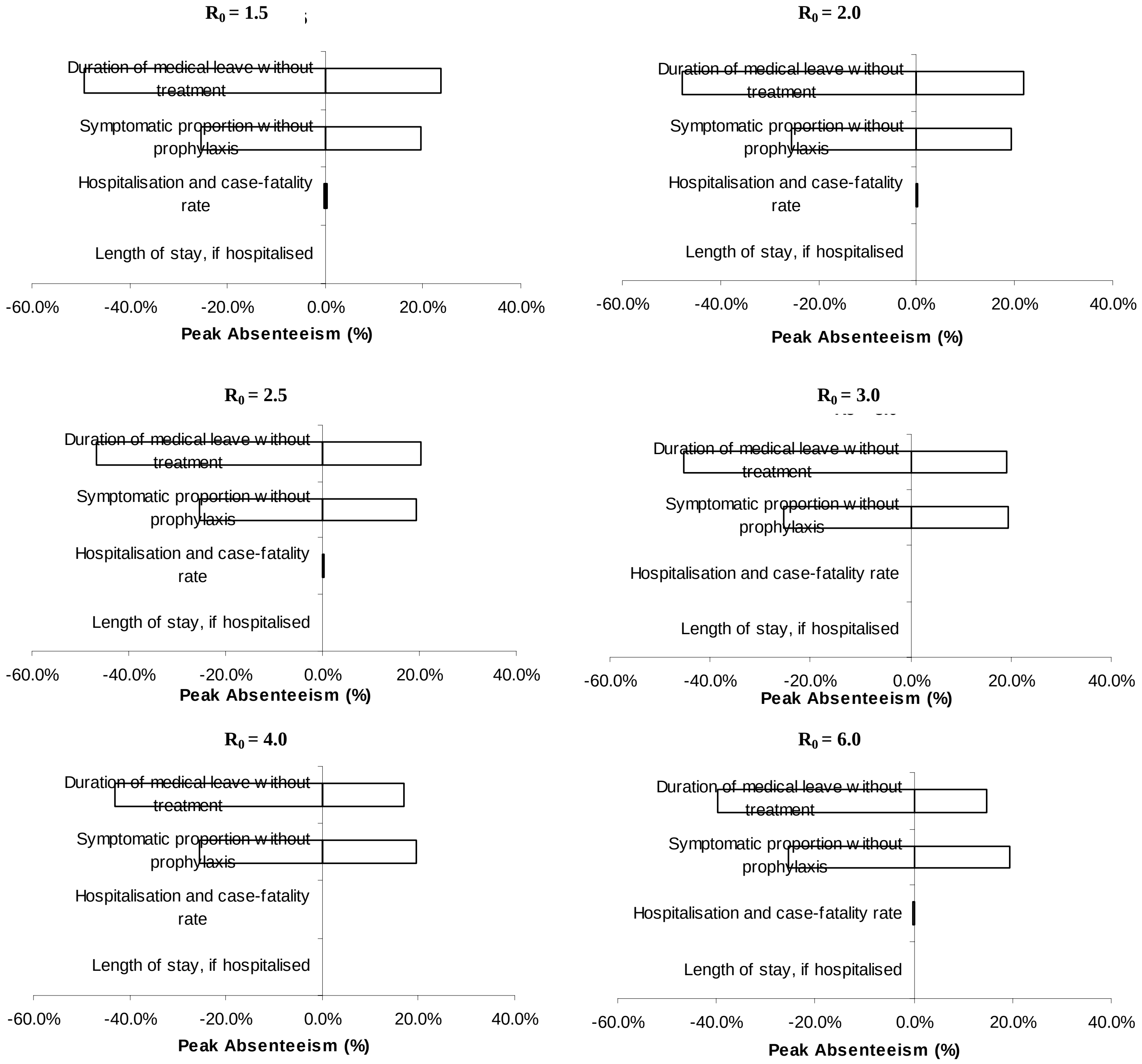

Figure A3. One-way sensitivity analysis for the strategy of treatment only, by $R_0$.

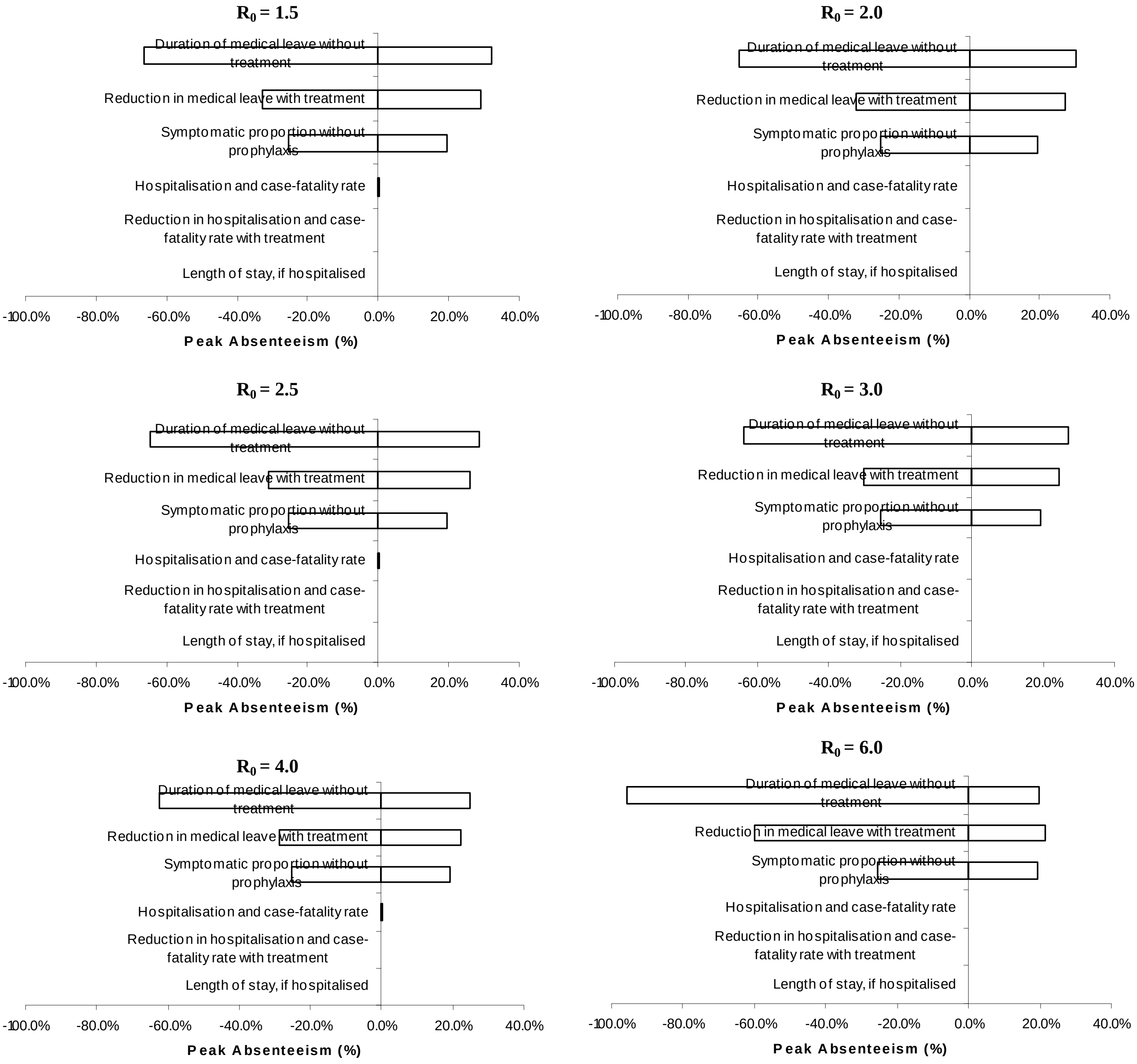

Figure A4. One-way sensitivity analysis for the strategy of prophylaxis, by R0.

**$R_0$ = 1.5**

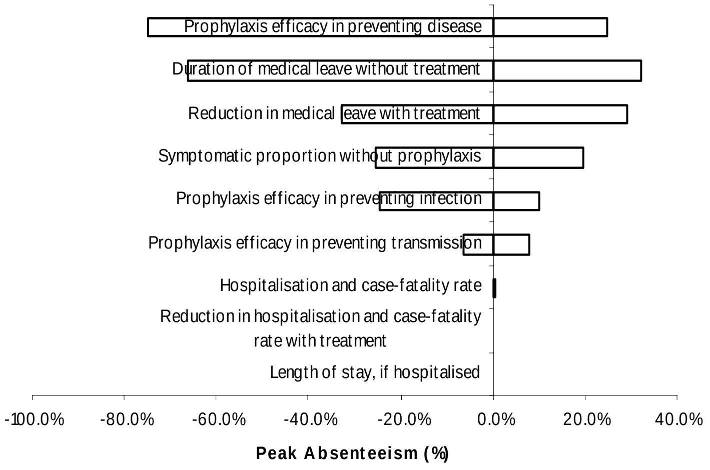


**$R_0$ =2.0**

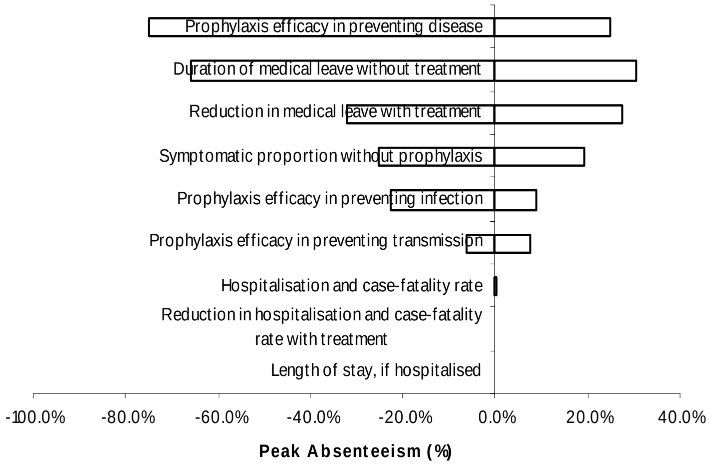


**$R_0$ = 2.5**

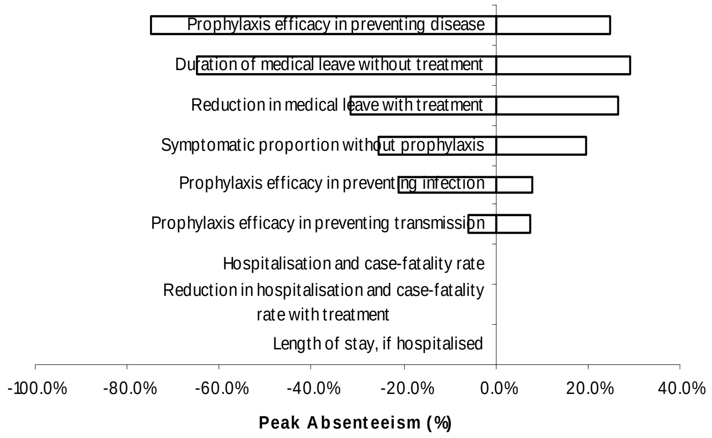


**$R_0$ =3.0**

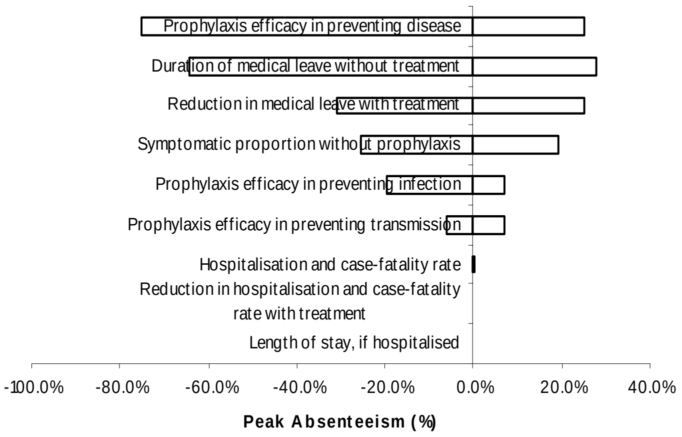


**$R_0$ = 4.0**

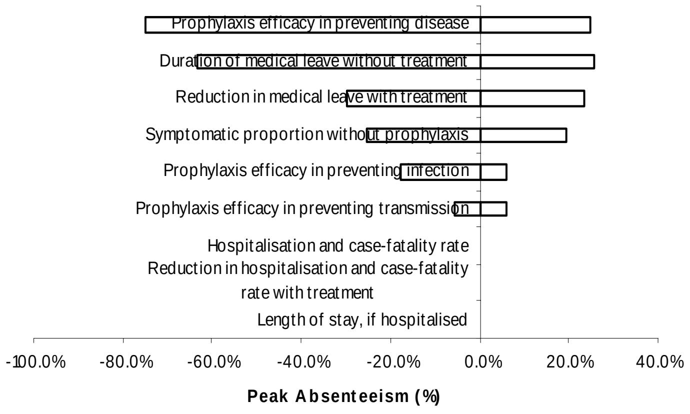


**$R_0$ = 6.0**

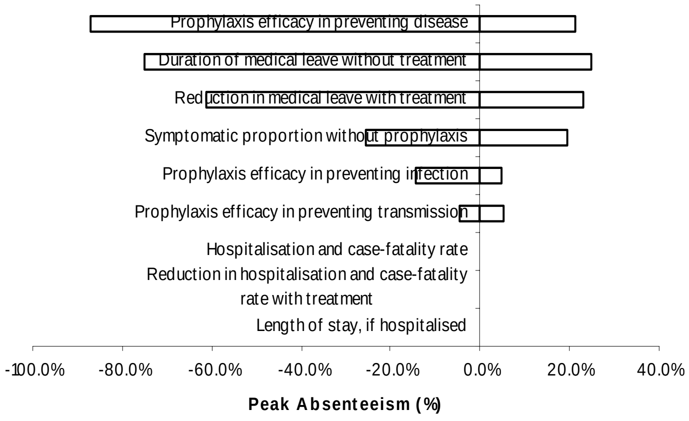

Figure A5. Peak absenteeism by treatment and prophylaxis strategies, H/P ratios, and HCW-to-HCW transmission ($\omega$), for $R_0$=1.5. (Tx refers to treatment, Rx refers to prphylaxis)

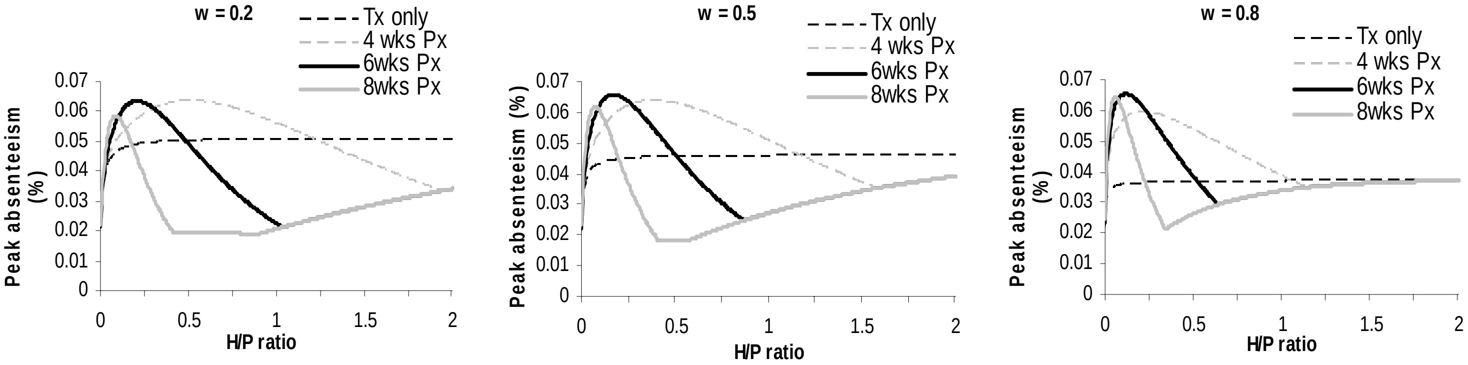


Figure A6. Peak absenteeism by treatment and prophylaxis strategies, H/P ratios, and HCW-to-HCW transmission ($\omega$), for $R_0$=2.0. (Tx refers to treatment, Rx refers to prphylaxis)

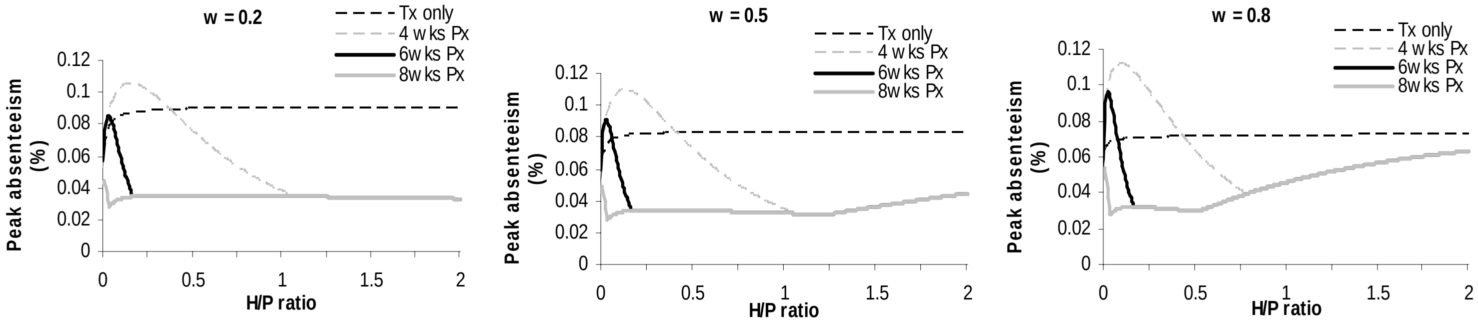


Figure A7. Peak absenteeism by treatment and prophylaxis strategies, H/P ratios, and HCW-to-HCW transmission ($\omega$), for $R_0$=2.5. (Tx refers to treatment, Rx refers to prphylaxis)

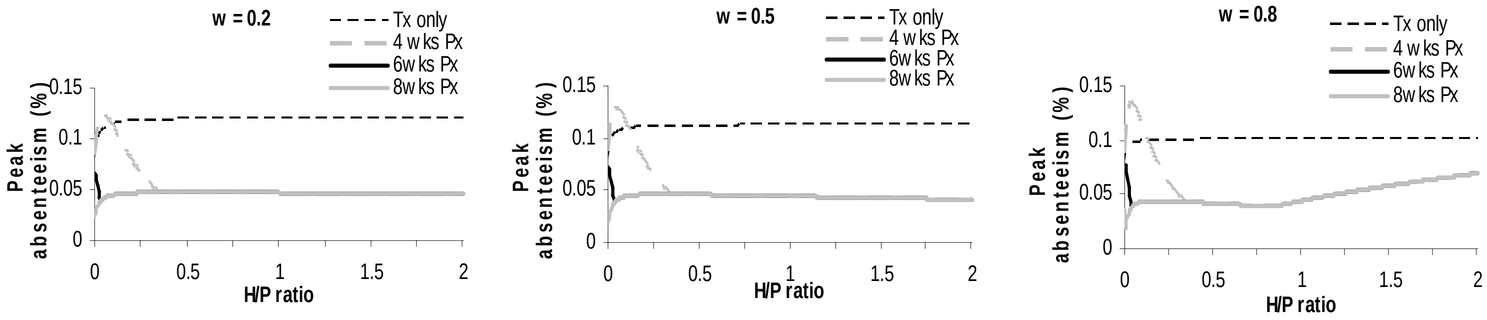

Figure A8. Peak absenteeism by treatment and prophylaxis strategies, H/P ratios, and HCW-to-HCW transmission ($\omega$), for $R_0$=3.0. (Tx refers to treatment, Rx refers to prphylaxis)

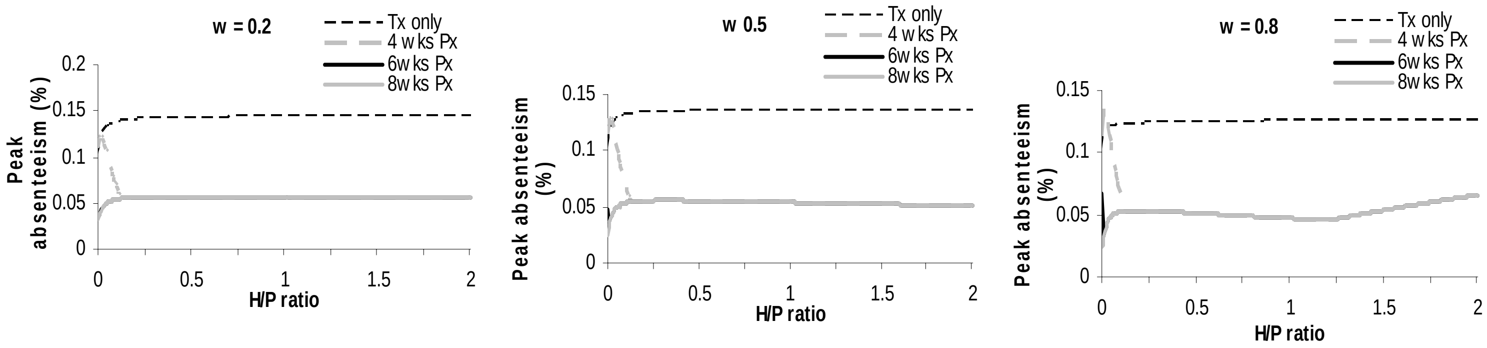


Figure A9. Peak absenteeism by treatment and prophylaxis strategies, H/P ratios, and HCW-to-HCW transmission ($\omega$), for $R_0$=4.0. (Tx refers to treatment, Rx refers to prphylaxis)

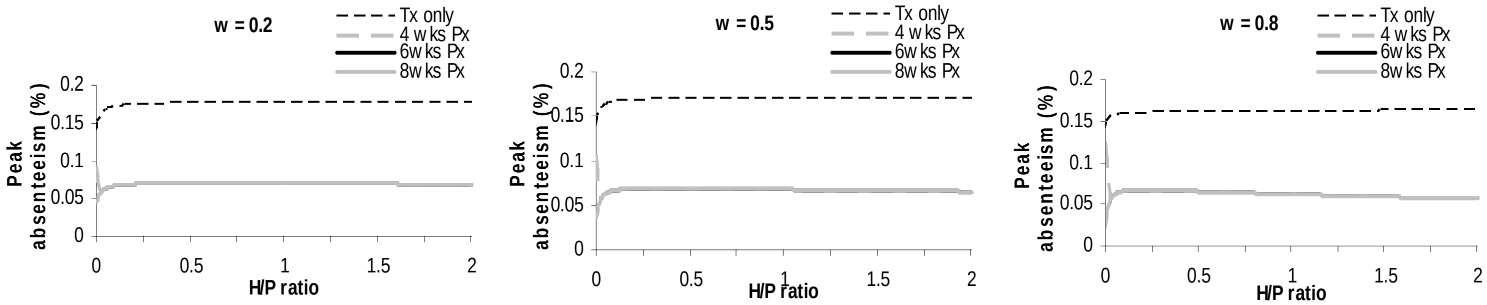


Figure A10. Peak absenteeism by treatment and prophylaxis strategies, H/P ratios, and HCW-to-HCW transmission ($\omega$), for $R_0$=6.0. (Tx refers to treatment, Rx refers to prphylaxis)

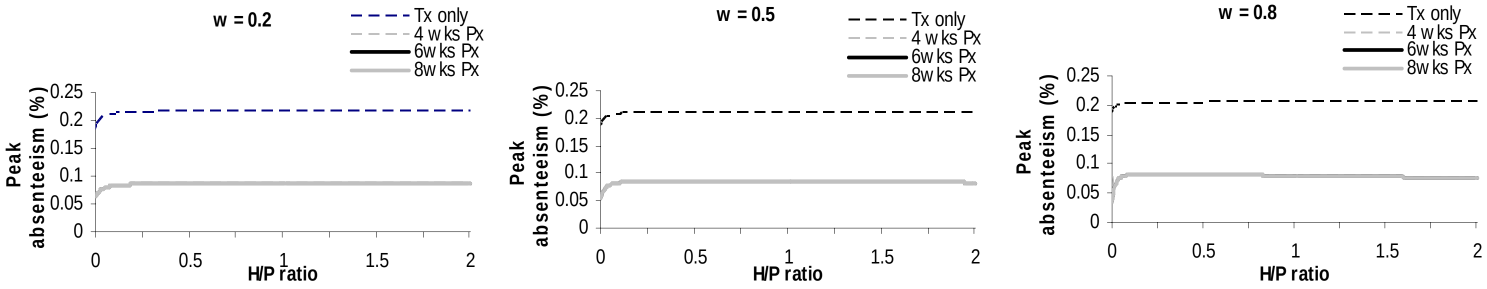

Supplement: Technical Appendix — Supplementary material including additional methodology, results, and discussion. [file 06-0309_Techapp-s2.pdf]
